# Alcohol’s Unique Effects on Cognition in Women: A 2020 (Re)view to Envision Future Research and Treatment

**DOI:** 10.35946/arcr.v40.2.03

**Published:** 2020-09-10

**Authors:** Rosemary Fama, Anne-Pascale Le Berre, Edith V. Sullivan

**Affiliations:** 1 Department of Psychiatry and Behavioral Sciences, Stanford University School of Medicine, Stanford, California; 2 Neuroscience Program, SRI International, Menlo Park, California

**Keywords:** alcohol, women, cognition, acute consumption, AUD, recovery

## Abstract

Alcohol use and misuse is increasing among women. Although the prevalence of drinking remains higher in men than women, the gender gap is narrowing. This narrative review focuses on the cognitive sequelae of alcohol consumption in women. Studies of acute alcohol effects on cognition indicate that women typically perform worse than men on tasks requiring divided attention, memory, and decision-making. Beneficial effects of moderate alcohol consumption on cognition have been reported; however, a number of studies have cautioned that other factors may be driving that association. Although chronic heavy drinking affects working memory, visuospatial abilities, balance, emotional processing, and social cognition in women and men, sex differences mark the severity and specific profile of functional deficits. The accelerated or compressed progression of alcohol-related problems and their consequences observed in women relative to men, referred to as “telescoping,” highlights sex differences in the pharmacokinetics, pharmacodynamics, cognitive, and psychological consequences of alcohol. Brain volume deficits affecting multiple systems, including frontolimbic and frontocerebellar networks, contribute to impairment. Taken together, sex-related differences highlight the complexity of this chronic disease in women and underscore the relevance of examining the roles of age, drinking patterns, duration of abstinence, medical history, and psychiatric comorbidities in defining and understanding alcohol-related cognitive impairment.

## INTRODUCTION

Alcohol use and misuse have increased among women over the past 2 decades,^1^ with an estimated 5.3 million women age 18 and older meeting criteria for alcohol use disorder (AUD) in the United States in 2018 (https://www.niaaa.nih.gov/alcohol-health/overview-alcohol-consumption/alcohol-use-disorders). The rate of AUD in women increased 84% over the past decade in comparison with a 35% increase in men.^2^ Although the prevalence of men who drink is still higher than that of women, the gender gap is narrowing.^2^–^4^ Of note, prevalence of drinking and binge drinking, defined by the National Institute on Alcohol Abuse and Alcoholism (NIAAA) as four or more alcoholic beverages on the same occasion for women, rose in older women (age 60 and older)^5^,^6^ compared with previously reported levels.

Commensurate with the rising rates of women with AUD should be enhanced efforts to examine sex differences related to consequences of alcohol consumption. Most of the earliest reports of the untoward consequences of alcohol focused on men and suffered from lack of statistical power to identify sex-related differences because of small numbers of female participants or unequal sample sizes between the sexes, raising limits on generalizability to women.^7^ Despite this bias, appreciation of sex differences in alcohol-related factors and consequences is not new. Indeed, Lisansky addressed the importance of examining alcohol factors uniquely related to women more than a half century ago.^8^ What is new, however, is greater insistence in research studies and clinical applications for systematic investigations to address sex-related differences in alcohol consumption, antecedent factors of drinking, and alcohol-related consequences. As a result of this mandate, work over the past decade has made it amply apparent that men and women differ in alcohol-related risks, health and cognitive consequences, and factors related to successful abstinence and sobriety.^9^

This narrative review focuses on the cognitive sequelae of alcohol use in women, including deficits associated with acute consumption, moderate drinking, at-risk or hazardous drinking, and chronic excessive drinking. (See the box **Effects of Alcohol Consumption on Women and Factors That Influence Research Outcomes**.) Over the years, nomenclature regarding alcohol misuse has changed based on scientific understanding of the disease—for example, “alcohol abuse” and “alcohol dependence” in the fourth edition of the *Diagnostic and Statistical Manual of Mental Disorders* (DSM-IV) evolved into “alcohol use disorder” by the fifth edition (DSM-5). Although anachronistic for studies predating DSM-5 nomenclature, the term “AUD” is used throughout this review when referring to individuals who met criteria for an alcohol misuse-related diagnosis at the time of assessment.

## SEX DIFFERENCES IN ALCOHOL METABOLISM AND THE CONSTRUCT OF “TELESCOPING”

Alcohol is metabolized at different rates in men and women,^10^ and these sex differences in the pharmacokinetics of alcohol are biologically founded. Particularly notable is sexual dimorphism of body composition. Compared with men, women generally have less body water and a higher proportion of fat, which does not absorb alcohol, resulting in higher blood alcohol concentration (BAC) levels, even when the amount of alcohol consumed is adjusted for body weight. In addition, women tend to have lower levels of gastric alcohol dehydrogenase, the enzyme that breaks down ethanol into its metabolites. Thus, BAC levels rise faster and stay elevated longer in women than men.^3^ It has been speculated that these sex-related pharmacokinetic differences underlie why women can develop health-related consequences, including cirrhosis of the liver, earlier in their disease and after lower total lifetime alcohol consumption than men.^7^,^11^

“Telescoping” describes the accelerated or compressed progression of the landmark events of AUD (e.g., age at first drink, age when started having problems related to alcohol, age when first entered treatment) in women compared with men.^12^,^13^ Initial studies addressing telescoping focused on duration of time from onset of drinking to time to enter alcohol treatment or time to develop medical problems (e.g., hepatic disease). Early studies reported that women initiate hazardous drinking—drinking that puts a person at heightened risk of developing AUD—at a later age than men, although they enter alcohol treatment earlier in their disease than men.^14^,^15^ Women also were reported to be more susceptible and to experience alcohol-related medical problems after a shorter time of chronic heavy drinking^12^ and lower lifetime consumption compared with men.^16^ Indeed, there is evidence that women are at heightened risk of alcohol-related heart disease.^3^ Taken together, there is increasing support for this phenomenon as it pertains to the physiological and health-related consequences of alcohol in women.^3^,^17^

Telescoping has been invoked in studies examining the timing and severity of cognitive deficits associated with chronic heavy drinking in women compared with men.^7^,^18^ Demonstration of a shorter duration from drinking to detectable cognitive deficits in women, however, has received mixed support, with some studies supporting the concept of telescoping of select cognitive processes,^18^ whereas other studies do not.^19^,^20^ Additional research is needed to examine the temporal sequencing, pattern, and severity of cognitive deficits in women and men in relation to landmark events associated with alcohol consumption. Inconsistency among studies examining the temporal sequence of events related to AUD in men and women could be due in part to methodological or even geographical factors, including accuracy of self-report and factors that mediate and moderate a woman’s decision to seek sobriety-related or health-related treatment, such as ease or availability of treatment and help with family responsibilities.^21^

## ALCOHOL’S EFFECTS ON COGNITION IN WOMEN

### Acute Alcohol Consumption

An early study directly compared the acute effects of alcohol on men and women who were social drinkers without an alcohol misuse diagnosis and reported that, after moderate levels of alcohol consumption (BAC = .04%), women scored lower than men on a short-term memory task.^22^ In a study examining divided attention and balance (sway) in light drinkers (12 men—average absolute ethanol intake in the 30 days prior to testing was 7.9 g/kg (range: 5.6–10.0 g/kg), 12 women—7.38 g/kg (range: 5.01–10.23 g/kg); ages 18 to 24), it was reported that the women scored significantly lower on divided attention than the men only at higher alcohol levels (BAC = .06%) and not lower levels (BAC = .03%) or for placebo.^23^ Sex-related differences were not observed in sway at any BAC level. Data summarized from seven experiments examining the effects of moderate alcohol dose (0.65 g/kg) in participants with no self-reported history of substance use disorder (ages 21 to 35) on driving performance indicated that these young social drinking women showed greater deficits in memory recall, divided attention, and motor skills than did young social drinking men who did not have AUD.^24^ In that review, all driving-related measures were impaired for both men and women after alcohol consumption compared with their nondrinking performance, with women demonstrating a larger decline in performance after drinking than men. These studies provide support for the notion that women may be more vulnerable than men to the cognitive effects of acute intoxication.^16^

By contrast, other studies have failed to find sex differences in relation to acute alcohol consumption. Accordingly, a study assessing 11 men and 13 women found no significant sex differences in performance on cognitive tests including assessment of divided attention, short-term memory, and rotary pursuit at moderate levels of acute consumption, blood alcohol levels (BALs) of .054% for men and .062% for women. BALs were measured at 20-minute intervals after the first drink by using a gas chromatographic intoximeter, and BALs were statistically controlled for in between-group analyses.^25^ Additionally, although both men and women were impaired, no sex differences were reported in a study that assessed flight simulation performance in general aviation pilots ages 21 to 40 at moderately high BALs (12 women = .084%, 11 men = .087%), levels exceeding legal limits of intoxication in the United States (BAL = .08%).^26^

Age can moderate the effects of acute alcohol consumption on cognition.^27^,^28^ A double-blind, placebo-controlled factorial design study assessing psychomotor, set-shifting, and working memory processes in community-dwelling social drinkers who had never met criteria for an alcohol misuse diagnosis (15 men, 24 women; ages 55 to 70) at low (breath alcohol concentration [BrAC] = .04%) and moderate (BrAC = .065%) levels of acute alcohol administration reported age-related deficits compared with 51 younger community-dwelling moderate drinkers (31 men, 20 women; ages 25 to 35). Both the younger and older adult groups exhibited some beneficial effect of low-dose alcohol compared with placebo on a simple psychomotor sequencing task (Trail Making Test, Part A). At the higher dose level (BrAC = .065%), however, only the older adults were impaired on a more complex psychomotor task requiring sequencing and working memory (Trail Making Test, Part B).^28^ Cognitive efficiency, the ability to perform quickly and accurately, was most compromised in the moderate-dosage group of older adults, regardless of sex.^28^

An examination of acute alcohol effects on cognition failed to identify sex differences in tests of set shifting, psychomotor speed, or working memory in non-problem drinking older adults (26 men, 36 women; ages 55 to 70) randomly assigned to one of three dose conditions: placebo; low dose (BrAC = .040%); and moderate dose (BrAC = .065%).^29^ The authors concluded that sub-intoxicating doses of alcohol do not differentially affect healthy, older, moderate-drinking men and women.

Taken together, studies that find sex-related differences on cognitive effects of acute alcohol consumption report that women tended to perform worse than men on higher-order cognitive tasks requiring divided attention, working memory, and decision-making, as opposed to less complex tasks such as reaction time or psychomotor measures.^9^ Inconsistency of findings across studies is likely due to a number of factors including subject selection, task demands, and heterogeneity of response to alcohol.

### Acute Cognitive Effects of Binge Drinking and Blackouts

Binge drinking can produce blackouts, defined by periods of amnesia (the inability to transfer information from short-term to long-term memory) experienced while an individual is apparently conscious and able to engage in activities such as walking, talking, and driving.^30^–^32^ Rapid increase of BAC is a major risk factor for a blackout, with BAC levels of .22% having upward of a 50% chance of producing a blackout.^33^ In young adults, blackouts are a common consequence of binge drinking.^34^ Of 2,140 young adults 1 year post high school, 68% reported consuming alcohol at some point in their lifetime, and 20% of that group reported a blackout in the past 6 months.^34^ The occurrence of blackouts was as prevalent among young women (17%) as men (22%) in this cohort. Blackouts have been associated with poor decision-making and impulsivity, and they increase the vulnerability of both women and men to unlawful, regrettable, and dangerous interpersonal and social situations. It has been speculated that blackouts could be more predictive than level of consumption of alcohol-related harms.^34^

### AUD and Chronic Excessive Consumption

DSM-5 conceptualizes AUD as a chronic relapsing disease, where an individual continues to drink despite knowing that one’s current drinking pattern is likely to lead to untoward medical, personal, and social consequences.^35^ The diagnosis of AUD is based on a severity continuum ranging from mild to moderate to severe, depending on the number of diagnostic criteria met, which include but are not limited to drinking more than intended, having difficulty refraining from drinking, drinking that interferes with work and family responsibilities, cravings, tolerance, and withdrawal. The AUD continuum differs from the previous diagnostic classification system, DSM-IV-TR,^36^ which made a categorical distinction between alcohol abuse and alcohol dependence. Studies investigating the effects of chronic heavy drinking on cognitive processes in women with an alcohol-related diagnosis defined by either DSM system often have reported deficits in line with those in men with an alcohol-related diagnosis, but a number of studies also have reported differences in the cognitive effects of alcohol based on sex, described next.^37^–^39^

Based on rigorous, quantitative assessments, cognitive deficits associated with chronic heavy drinking in women have been reported since the early 1980s.^19^,^40^ One of the earliest studies compared 33 recently sober women (10 to 23 days since last drink) with 44 age- and education-matched control women on a number of cognitive and motor domains. Impairments were observed in visuospatial processing (block design), psychomotor speed (trail making), information processing (digit symbol substitution), and memory (verbal and visual recognition and recall).^18^ The authors of this study noted that the women with AUD displayed significant cognitive and motor deficits, yet had a notably shorter drinking history than participants in previously reported studies that included men with AUD.^18^ Indeed, even after statistically controlling for differences in drinking histories between men and women—duration of hazardous drinking in men was more than twice that of women (13 years vs. 6 years, respectively)—and then separately matching men and women on age and years of problem drinking, the study found that women still scored significantly lower than men on tests of memory recall and psychomotor speed.^14^ However, it has been cautioned that, given the cross-sectional nature of the study, it could not be determined whether cognitive deficits in the women were a risk factor for or a consequence of drinking.^14^

The pattern and extent of cognitive and motor deficits across six domains (i.e., executive functions, short-term memory and fluency, declarative memory, visuospatial abilities, upper-limb motor ability, postural stability) were examined in 43 recently sober (average duration, 3.6 months; range 2 to 15 months) women with AUD ages 28 to 63.^41^ Compared with 47 no- to low-drinking control women matched on education and scores standardized on age, the women with AUD demonstrated deficits in verbal and nonverbal working memory, visuospatial abilities, and postural stability (balance and gait), with relative sparing of executive functions, declarative memory, and upper limb strength and speed.^41^ By comparison, an earlier study examining the pattern and extent of cognitive deficits in 71 recently (1 month) sober men with AUD—compared with 74 healthy control men—reported deficits in executive function, visuospatial abilities, and gait and balance in men with AUD.^42^ Taken together, these studies demonstrated that both women and men with AUD showed impairment on visuospatial processes; however, compared with nondrinking, sex-matched control participants, only the women were impaired on tasks of short-term memory, and only the men exhibited executive function deficits.

In a more recent cross-sectional study of 164 older DSM-IV alcohol-dependent participants (62 women, 102 men; age 62.6 ± 6.4 years), women performed better than men on mental flexibility as assessed by the Trail Making Test.^43^ By contrast, men performed better than women on a test of visual processing assessed with a figure recognition task. Despite impairment in men and women, sex differences were not forthcoming on ability to overcome cognitive interference assessed with the Stroop Color and Word Test.^43^

Taken together, chronic excessive drinking in women is associated with myriad cognitive deficits, overlapping but not identical to the pattern of deficits observed in men. Although some evidence indicates that women develop cognitive deficits earlier in their disease or at lower lifetime consumption rates than men, its generalizability has not been clearly established.

## POTENTIAL BENEFITS ASSOCIATED WITH MODERATE DRINKING

Despite the association of chronic excessive drinking with cognitive and motor deficits, much has been made about the potential beneficial health effects associated with moderate drinking—notably decreased risk of cardiovascular disease, better overall cognitive ability, and a slower rate of cognitive decline associated with normal aging.^44^–^47^ Moderate drinking is generally defined as no more than one standard drink (14 grams of 95% alcohol) per day for women and two standard drinks per day for men. The pattern of performance from no drinking to excessive drinking has often been denoted as a U-shaped curve^48^,^49^ or a J-shaped curve^50^ with amount drunk modifying performance level.

Even moderate levels of alcohol consumption, however, have been associated with an increased risk of breast cancer, liver-related diseases, and cardiomyopathy in women (https://www.niaaa.nih.gov/publications/brochures-and-fact-sheets/women-and-alcohol), as well as infectious diseases, gastrointestinal disorders, and alcohol-related injuries.^51^ In addition, for older women (particularly those age 60 and older), interactions between alcohol consumption at any level and aging, age-related disease, and drugs commonly prescribed to older people (including antibiotics, antidepressants, anxiolytics, and warfarin) can be hazardous.^52^ Indeed, in addition to comorbid use of other drugs and medical comorbidities, AUD in older women often presents with complex clinical issues including untreated or undertreated depression and anxiety, which can exacerbate problems related to consumption and consequences of alcohol, family responsibilities, and feelings of guilt and shame surrounding their drinking. Although concern for older women in relation to alcohol consumption is not new,^53^ there remains a dearth of literature addressing the complexity of the factors associated with AUD in the elderly. With such a range of medical and mental health problems in this subpopulation, personalized treatment plans taking into account the entire picture and not just problem drinking are needed if abstinence and recovery are to be successful.^52^

An early study examining sex differences in 1,389 low to moderate drinkers (574 men, 815 women; ages 59 to 71) reported that women who were light (fewer than two drinks daily) to moderate (two or three but fewer than four drinks daily) drinkers performed better on set shifting, as assessed by the Trail Making Test, Part B, than women who reported abstaining from alcohol.^48^ This beneficial effect of light to moderate drinking was not observed for men. These authors reiterated the importance of controlling for variables such as age, education, income, depressive symptoms, and smoking status in studies examining sex-related cognitive differences in relation to alcohol.

More recently, a longitudinal study of 818 older adults (age 65 and older; 139 moderate drinkers and 679 nondrinkers) found that although moderate alcohol use (defined as one to 14 drinks per week; average number of drinks per week in this cohort = 5.02 ± 3.79 *SD*) was related to higher baseline cognitive performance, no relation was observed on rate of change over time (spanning 7 years) across cognitive domains.^54^ These authors highlighted the importance of future research focusing on the influence of demographic, genetic, and lifestyle factors on the variability observed in moderate drinking in relation to cognition. Indeed, another study cautioned that studies reporting beneficial effects of moderate drinking may have included an inappropriate selection of reference groups and little control for confounders.^55^ The authors of this study found a beneficial dose-response relation only for women drinkers age 65 and older, with no measurable benefit of moderate drinking in other age-sex groups.

Another longitudinal study examined the relation between cognitively healthy longevity—defined as living to age 85 without cognitive impairment, as assessed by the Mini-Mental State Examination—and amount and frequency of alcohol intake in 1,344 older community-dwelling adults (728 women and 616 men; ages 55 to 84) and found a beneficial effect of regular, moderate drinking.^44^ Indeed, individuals who reported drinking at moderate to heavy levels—up to three standard drinks per day for women on a near-daily basis—had twofold higher odds of living to age 85 without cognitive impairment compared with nondrinkers.^44^ Nonetheless, another study of nondemented autonomously living octogenarians reported that older women who drank moderately did not appear to benefit at the same level as older men who drank moderately when it came to cognitive performance.^56^ Indeed, only a relatively modest benefit in verbal memory for short stories was observed in women compared with men with moderate-level drinking. Sex differences were speculated to be due to myriad factors including drinking patterns and alcohol-related pharmacokinetics.

## ALCOHOL CONSUMPTION AND RISK OF DEMENTIA

It is projected that the U.S. population age 65 and older will nearly double, from 48 million currently to 88 million by 2050 (https://www.nih.gov/news-events/news-releases/worlds-older-population-grows-dramatically). With an ever-increasing aging population, it is imperative to understand the effects of chronic excessive drinking on the structure and function of the aging brain and the moderating and mediating effects of age-related medical and psychiatric conditions, interactions with medications, and life-related stressors.

A meta-analytic study assessing risk of dementia in relation to alcohol consumption reported a modest U-shaped relation.^57^ Results highlighted that moderate alcohol consumption, defined as fewer than 12.5 g/day (about one standard drink), was associated with a reduced risk of dementia, whereas drinking to excess (defined as ≥ 23 standard drinks per week) was associated with a significantly greater risk of dementia compared with light drinking. The lowest risk of dementia was associated with drinking 6 g/day of alcohol, and wine was reported to be selectively associated with protective effects.

Another study—which included 2,874 women (of 9,087 total participants) with an average length of follow-up of 23 years—reported that abstainers and those who drank heavily (defined as more than 14 standard drinks per week) had a greater risk of dementia, determined from electronic health records.^58^ These authors speculated that nondrinkers and those who drink excessively may be at higher risk of cardiometabolic disease including diabetes and hypertension, which, in turn, is associated with an increased risk of dementia.

At-risk drinking in the elderly is a timely issue. One study noted that 12% of older women (age 60 and older) reported drinking in excess of the recommended guidelines of no more than one standard drink a day or seven standard drinks per week but without meeting diagnostic criteria for AUD.^52^ Without proper screening and intervention, these older adult women may be at particular risk for alcohol-related health and cognitive problems including dementia.

## EMOTIONAL PROCESSING AND SOCIAL COGNITION IN WOMEN WITH AUD

Over the past decade, emotional processing and social cognition have become a focus of addiction research, highlighting the relevance of one’s abilities to identify and respond to emotional and social cues in interpersonal interactions at home, at work, and with friends. Sex differences outside of AUD typically note better performance in women than men in decoding emotional facial expression and in performing tasks of social cognition such as the Reading the Mind in the Eyes Test or the Faux Pas Recognition Test.^59^–^63^ Taken together, these findings suggest a potential resilience to social cognition disorders in women. This section reviews whether AUD disrupts this protective factor as a whole or interferes with selective processes.

AUD is associated with difficulties in components of emotion processing and social cognition, notably alexithymia, issues in decoding others’ emotions, inferring others’ mental states or feelings (i.e., Theory of Mind [ToM] deficit), and experiencing empathy.^64^ Factors contributing to deficits in emotional processing and social cognition include an increased risk of personal, social, and work problems as well as poor initiation of action to achieve abstinence in AUD.^65^ Vulnerability to emotional decoding and social cognition impairment in women with AUD may trigger an additional burden in their emotional and interpersonal interactions, thereby increasing relapse risk. Despite known sex differences in the severity of brain compromise and cognitive impairment in AUD,^66^ the literature on sex differences in emotional processing and social cognition in AUD is scant.

Alexithymia is a multidimensional personality construct that comprises four core characteristics: (1) difficulty identifying feelings in oneself and differentiating feelings from the physical sensation of emotional arousal, (2) difficulty describing feelings to others, (3) restricted imaginative processes featured by limited fantasy life, and (4) an externally oriented style of thinking.^67^ Alexithymia is commonly assessed by the Toronto Alexithymia Scale-20 (TAS-20), a self-report questionnaire, exploring three factors: difficulty identifying feelings, difficulty describing feelings, and externally oriented thinking (i.e., tendency to focus attention outside of oneself).^68^ Higher prevalence of alexithymia in women with AUD than in men with AUD has been observed, especially on the global TAS-20 score and its “difficulty identifying feelings” factor.^69^ Interestingly, alexithymia factors can play a moderator role in the relations between depressive mood and craving for alcohol in recently detoxified individuals with AUD.^70^ In particular, women with AUD who reported difficulty describing feelings were at higher risk for craving when experiencing depressed mood, which is consistent with the hypothesis that relapse would be more frequently associated with negative affect in women than men.^71^

Emotion decoding skills are crucial when assessing one’s immediate social environment, providing valuable information regarding others’ internal affective state, enabling behavioral adaptation according to others’ thoughts and intentions, and facilitating social interactions in daily life. Contradictory findings on sex differences have been reported in studies that assessed decoding of emotional facial expressions (EFE) in AUD. Although no evidence of sex differences was found in recently detoxified individuals,^72^,^73^ vulnerability to alcohol-related EFE recognition deficits was reported in recently detoxified women.^74^,^75^ Lack of consistency between studies could be related to the small sample sizes of women (fewer than 15 women), which may not be representative of the population of women with AUD. Elsewhere, assessment with the social cognition module of the Wechsler Advanced Clinical Solutions revealed significant impairment in recognizing affect from facial expression in long-term abstinent men but not in long-term abstinent women.^76^ Although the women did not differ from their sex-matched controls, better identification of emotional facial expressions was related to longer length of abstinence.

ToM refers to the ability to attribute mental states to oneself and others, and to understand that others’ mental states might differ from those of oneself.^77^ ToM enables individuals to predict, anticipate, and interpret the behavior of others and facilitates appropriate social interactions.^78^ Large effect sizes were identified in two recent meta-analyses for deficits in ToM in AUD.^79^,^80^ In support of the vulnerability hypothesis of emotional and social functioning impairment in women with AUD, a meta-analysis indicated that the effect size was modulated by sex, such that increasing the percentage of men in the treatment group decreased the effect size—results suggesting that “AUD is more likely to be associated with affective ToM deficits in females.”^80^^(p 413)^

## SEX DIFFERENCES IN ALCOHOL EFFECTS ON BRAIN STRUCTURE AND FUNCTION

Three decades of magnetic resonance imaging (MRI) studies describe patterns of brain structural abnormalities characteristic of chronic, heavy drinking.^81^,^82^ Despite the rich literature on neuroimaging in AUD, the mainstay of studies does not address sex differences. The focus of this section is on the research in women with AUD and starts with studies using conventional structural MRI to quantify regional brain volumes; also summarized are studies using magnetic resonance diffusion tensor imaging to assess the microstructural integrity of white matter fibers and finally functional MRI done in the task activation state.

### Structural MRI

Individuals with AUD but without neurological complications generally show ventricular expansion and shrinkage of selective cerebellar lobules and regions of the cerebral cortex. Volume deficits in cerebellar and cortical regions generally extend to gray and white matter macrostructure and microstructure. Whole-brain analyses support the profile of widespread damage to gray matter structures, including the frontal cortex, thalamus, insula, hippocampus, and cerebellum, as well as white matter regions including the cerebellar peduncles, pons, corpus callosum, and periventricular area.^83^–^87^ The exploration of specific brain damage in women with AUD has been limited by an inclusion bias of men in most studies and by the lack of methodological consideration of sex differences with respect to an appropriate control group matched in sex and other relevant factors to the clinical group. The few neuroimaging studies considering differences between men and women on alcohol-related brain structural changes have generated conflicting results.

A number of cross-sectional studies investigating brain macrostructural abnormalities and alcohol misuse have reported no sex differences in brain volumes.^85^,^88^ However, other studies have reported inconsistent findings including greater vulnerability in men than women,^89^,^90^ greater susceptibility to structural abnormalities in women than men,^91^,^92^ and sex-related differences in the pattern and severity of regional brain volumetric deficits.^66^ A study using a longitudinal design tested for, but did not find, sex differences on brain volumes related to chronic heavy drinking.^93^

Hippocampal volume deficits were identified in individuals with moderate alcohol consumption (fewer than 14 standard drinks per week for women, fewer than 21 standard drinks per week for men) in a study of 527 community-dwelling men and women who did not have AUD (mean age = 43 ± 5.4 years). This dose-dependent relation between alcohol consumption (i.e., alcohol units/week) over 30 years and hippocampal shrinkage, however, was significant only for men and not for women.^49^ A lack of effect in women may be attributed to inadequate statistical power given the smaller number of women (*n* = 103) than men (*n* = 424) in the study and the fact that few women in the study were categorized as unsafe drinkers (*n* = 14 women reported drinking more than 14 standard drinks per week). In addition, no demonstrable beneficial effect was observed with light alcohol consumption compared with abstinence on brain structure and function. The authors cautioned that the protective effect reported in association with moderate drinking in other studies may be due to confounding variables, such as socioeconomic status or IQ. Beneficial effects, defined as a reduction of age-related decline in brain volume, also were not observed in a study of nondependent (DSM-IV) drinking men and women, with a relation between greater amount of alcohol consumed and smaller total brain volume, which was more pronounced in women than men.^94^

### Diffusion Tensor Imaging (DTI)

This neuroimaging approach enables examination of the integrity of the microstructure of white matter, which comprises linearly organized fiber tracts that connect proximal and distal gray matter regions (that is, brain structures composed of neurons). Fiber integrity is measured in terms of fractional anisotropy (FA), typically higher in fibers with a homogeneous or linear structure such as healthy white matter, and bulk mean diffusivity of water movement for which higher values reflect diminished integrity or edematous tissue. In men with AUD, the greatest microstructural white matter abnormalities are reported in the corpus callosum, but for women with AUD, these abnormalities are greatest in the centrum semiovale.^95^ In other cross-sectional DTI studies, when matched for alcohol history variables, women with AUD showed more signs of white matter degradation than men with AUD in several fiber bundles, suggesting an enhanced risk for alcohol-related degradation in selective white matter systems.^96^ By contrast, no evidence for alcohol-related sex differences was forthcoming in DTI metrics for six anatomically defined transcallosal white matter fiber bundles.^97^

Potential sex differences in brain structural recovery with abstinence require further investigation. Contradictory results based on relations with length of abstinence^66^,^98^ showed stronger positive association between length of sobriety and white matter volumes in women with AUD than in men with AUD within the first year of abstinence.^66^ By contrast, positive associations between length of sobriety and white matter volumes were observed in men with AUD but not in women with AUD after 1 year of abstinence, suggesting faster white matter recovery in women.

Another DTI study reported relations between longer duration of abstinence and higher FA of the callosal white matter in men with AUD, but not in women with AUD.^98^ The authors suggested better callosal white matter recovery with abstinence in men, especially when men with shorter length of abstinence showed lower FA than recently abstinent women, but the opposite pattern was observed for longer duration of abstinence. Moreover, recent neuroimaging investigations found sex interactions displaying opposite patterns. Compared with control men, men with AUD had smaller volumes in the reward network and lower FA in select white matter tracts. By contrast, women with AUD had larger volumes in the reward system and higher FA in the same white matter tracts compared with control women.^98^–^100^ These authors suggested that this opposite pattern in brain structural abnormalities between men and women with AUD might reflect a sex-specific phenotype related to dissimilarities in neuroanatomical and neurobehavioral expressions as risk factors or in sex-based motivation to seek alcohol.

### Functional MRI

The literature investigating sex-related effects on brain functioning in AUD with functional MRI (fMRI) is scarce and is sampled next. A task-activated fMRI study revealed lower brain activation in the prefrontal and parietal cortices during a spatial working memory task in 10 women with AUD compared to 10 healthy women controls.^101^ During high-risk decisions to drink, control women activated the default mode network, whereas women with AUD simultaneously activated the reward, cognitive control, and default mode networks. These results suggest that risky decisions to drink could be associated with difficulties to switch between different neural networks in women with AUD, potentially due to dysfunction in the anterior insula.^102^

A small fMRI study of airplane pilots—individuals with AUD (8 women, 6 men) and healthy controls (9 women, 5 men)—revealed an interactive effect of AUD and sex on brain activation during negative and positive facial affective processing, such that men with AUD demonstrated higher brain activation than control men, whereas women with AUD showed lower brain activation than control women.^103^ By contrast, an fMRI study conducted in long-term abstinent individuals with AUD reported sex-related differences in the pattern of brain responsivity to emotional stimuli, with lower activation in the rostral middle and superior frontal cortex, precentral gyrus, and inferior parietal cortex in men with AUD than in control men, whereas higher activation in superior frontal and supramarginal cortices were observed in women with AUD compared to control women.^104^ As suggested, these specificities in brain reactivity between men and women during emotional processing may reflect sex-related differences in the emotional mechanisms leading to the development of AUD.

Taken together, these studies demonstrate the relation between chronic heavy drinking and structural and functional brain abnormalities in men and women; however, due to their cross-sectional nature, these studies cannot determine whether AUD-related brain dysmorphology was caused by drinking, was pre-existing, or both. Prospective longitudinal studies—such as the National Institutes of Health/NIAAA-supported National Consortium on Alcohol and Neurodevelopment in Adolescence (NCANDA)^105^ and the Collaborative Studies on the Genetics of Alcoholism (COGA)^106^—study adolescents before they initiate appreciable drinking. Assessing children as young as age 8, the Adolescent Brain Cognitive Development (ABCD) Study is a longitudinal prospective study^107^ that aims to identify the antecedent and resultant effects of alcohol and to track the drinking patterns that contribute to deviations from normal neurodevelopmental growth trajectories in cerebral^108^ and cerebellar^109^ volumes starting in preadolescence. These studies also will provide information that can address questions of specific sex-related risk factors that contribute to excessive drinking behavior and underlie differential prodromal brain abnormalities between men and women with AUD.

## RECOVERY OF COGNITIVE ABILITIES WITH SUSTAINED ABSTINENCE

On an optimistic note, potential for recovery of selective cognitive deficits including memory and psychomotor abilities can occur with sustained abstinence. Functions that appear more resistant to recovery include visuospatial skills and gait and balance stability, which often endure even with long-term abstinence.^110^–^113^ Cognitive impairment has been associated with higher rate of relapse and lower motivation to initiate and maintain abstinence.^114^

One of the earliest studies examining recovery of cognitive function with abstinence included both short-term abstinent (1 month, *n* = 40) and long-term abstinent (4 years, *n* = 40) women*.*^115^ This study indicated differential recovery among cognitive processes, with long-term sober women showing improvement on complex tasks of abstraction, assessed with the Halstead Category Test, whereas perceptuomotor ability, assessed with the Digit Symbol Test and the Trail Making Test, Part A, was more resistant to recovery. Critically, it was the subset of women who resumed drinking after baseline assessment that accounted for the greatest deficits at baseline compared with the subset of alcoholic women who remained sober. These authors highlighted the possibility that heterogeneity within their cohort could partly be explained by difference in posttreatment drinking (resumers vs. abstainers) and by differential premorbid “at-risk” variables in women compared with men with AUD.

Follow-up of a cohort of women with AUD at 3 to 6 years post–baseline testing after an average of 3 months of sobriety^41^ reported recovery of nonverbal short-term memory and psychomotor speed.^111^ Postural instability, however, was still noted, even after this extended length of abstinence. These studies highlight the selectivity of dissociable cognitive and motor processes in terms of time course and extent of recovery with abstinence.

An investigation of cognitive recovery after 6-week sobriety in a controlled environment after being in a residential treatment unit reported that a slightly lower percentage of women than men (41% vs. 46%) showed recovery on a general cognitive measure.^116^ These authors speculated that the timeline of recovery and factors promoting recovery may differ between men and women and highlighted the relevance of examining the effect of sex on remediation and extent and the timeline of recovery of component cognitive processes.

## FACTORS THAT MODERATE OR MEDIATE COGNITIVE AND MOTOR PERFORMANCE IN WOMEN WITH AUD

Hormonal differences between men and women and within cohorts of women have been hypothesized to at least partially underlie sex differences reported in AUD, although studies to establish this relation have been inconsistent and inconclusive.^9^,^117^ Only limited evidence suggests that phase of menstrual cycle accounts for a significant amount of the variability in behavioral response to alcohol, with a number of studies finding that phase of menstrual cycle had no significant effects on alcohol consumption in women.^117^,^118^ In addition, no differences among menstrual phases in alcohol pharmacokinetics have been forthcoming.^119^

Other factors speculated to moderate or mediate cognitive performance between alcoholic men and women or to underlie the heterogeneity among women with AUD are (1) age and aging effects and their interaction with alcohol; (2) alcohol consumption variables including age of AUD onset, amount drunk in one’s lifetime, quantity and pattern of binge events, family history of alcohol misuse, and number and severity of withdrawals; (3) nutritional status including thiamine and other vitamin B deficiencies; (4) existence of comorbid medical and health conditions including HIV, hepatitis C, and chronic pain; (5) other drug use (including prescription and illicit); and (6) psychiatric symptoms and disorders.^37^,^65^,^120^

Research strongly supports the notion that whether one maintains sobriety or relapses into drinking, even when drinking does not meet AUD criteria, may moderate the extent and rate of cognitive and motor recovery in AUD. Attention has been paid recently to the history of trauma and chronic pain and their relation to initiation and maintenance of hazardous drinking in women and bidirectional effects of alcohol on these factors.^120^,^121^

Pain, for example, may be both a risk factor and a consequence of excessive drinking.^121^,^122^ Although alcohol can reduce and even quell pain in some individuals when alcohol is initially used, over time increasing amounts of alcohol are needed to achieve pain relief, with the paradoxical effect that alcohol consumption exacerbates pain intensity. In a study of 451 treatment-seeking participants with an alcohol misuse diagnosis in residential treatment, women were more likely to report significant recurrent pain, more concurrent chronic pain conditions, and greater pain severity than men.^122^ Taken together, these studies highlight the relevance of including effective pain management in initiation and maintenance of abstinence, particularly in women.

## LIMITATIONS OF STUDIES

Limitations commonly noted in studies on the cognitive effects associated with chronic excessive drinking include the fact that most of the data pertaining to alcohol consumption variables, including pattern, severity, and amount, are obtained through self-report. Structured follow-back interviews likely aid accuracy of documentation but are subject to memory distortion. Differences in subject inclusion and exclusion criteria and task demands make it difficult to generalize across studies; standardization of participant characteristics and tests would allow meta-analyses across data. Additionally, the dearth of longitudinal reports limits the ability to determine whether a deficit was pre-existing or caused by alcohol misuse or to document the temporal sequence of cognitive declines and recovery in relation to the dynamic nature of alcohol use.

Additional limitations relevant to review of studies on moderate alcohol consumption and cognition and women include inclusion of “sick quitters” in the group of abstainers—that is, individuals who no longer drink because of previous alcohol misuse.^51^ Efforts were taken to include studies where this was not a clear issue. Further, this review only included studies assessing sex differences and not gender differences, per se.

## TREATMENT IMPLICATIONS AND CONCLUSION

There is a growing appreciation of direct comparisons between men and women in the examination of alcohol’s effects on brain structure and function and the identification of factors contributing to alcohol-related cognitive impairment, including those that affect personal, social, and professional lives. Of course, regardless of sex, assessment of cognitive deficits is relevant to treatment plans, as it has been documented that efficacy of treatment with a heavy cognitive behavioral therapy component may be best delayed until recovery of the cognitive processes relevant to task demands.^123^

Highlighting the cognitive effects of acute, moderate, at-risk, and excessive drinking in women speaks to the urgency of screening, treating, and monitoring women who report patterns of possible alcohol misuse, even if diagnostic criteria for AUD are not met.^124^ Young adults should be educated on the cognitive effects of binge and intensive drinking for both the short term and the long term.^125^ Older adult women need to be educated on how alcohol interacts with age-related biological changes, comorbid medical conditions related to aging, and medications.

Longitudinal studies that examine the pattern and extent of cognitive and motor deficits associated with chronic heavy drinking and the factors that play a role in initiation and maintenance of alcohol misuse will continue to have both theoretical and clinical implications, steering specialized treatment for women with AUD and informing practice and policy. Heterogeneity among women with AUD highlights the complexity of this chronic disease and underscores the relevance of examining the effects of demographic factors, especially age and aging factors, and disease-related variables, notably pattern of drinking and duration of abstinence, in identifying the cognitive effects of alcohol and its biological underpinnings.

**Effects of Alcohol Consumption on Women and Factors That Influence Research Outcomes**

**Table:** 

What We Know	Factors That Influence Research Outcomes

Acute alcohol consumption	* Differences in task demands * Heterogeneity of response to alcohol * Small sample sizes * Differences in study inclusion and exclusion criteria * Cross-sectional vs. longitudinal study * Important to control for variables such as ♦ Age ♦ Education ♦ Socioeconomic status (SES) ♦ Depression/anxiety symptoms ♦ Smoking status ♦ Drinking patterns ♦ Alcohol-related pharmacokinetics ♦ Hormonal differences ♦ Nutritional status ♦ Comorbid medical conditions ▪ HIV ▪ Hepatitis C ▪ Non-alcohol substance misuse ▪ Psychiatric conditions ▪ Chronic pain

Deficits reported in women	
Divided attention Psychomotor speed Working memory Short-term memory Set-shifting Decision-making	

Moderate drinking	

Modest beneficial effects Better overall cognitive ability Slower rate of cognitive decline in aging Increased risk of Breast cancer Gastrointestinal disorders Infectious diseases	

Chronic excessive alcohol consumption	

Telescoping	
Compared with men: Women have shorter intervals between landmark events from the inception of drinking to entering treatment. Women experience medical and health-related problems earlier, even when duration and amount of alcohol consumed are comparable between the sexes. Women exhibit different patterns and severity of cognitive compromise, some modulated by sex-related emotional and social factors.	
